# Editorial: Advancing antimicrobial strategies: nucleic acid and peptide-based approaches

**DOI:** 10.3389/fmicb.2026.1807695

**Published:** 2026-03-03

**Authors:** Nuno F. Azevedo, Sybil Obuobi, Nhan Dai Thien Tram, Peter E. Nielsen

**Affiliations:** 1 LEPABE, ALiCE, Faculty of Engineering, University of Porto, Porto, Portugal; 2 Drug Transport and Delivery Research Group, The Department of Pharmacy, UiT The Arctic University of Norway, Tromsø, Norway; 3 Department of Pharmacy and Pharmaceutical Sciences, National University of Singapore, Singapore, Singapore; 4 Department of Cellular and Molecular Medicine, Kobenhavns Universitet, Copenhagen, Denmark

**Keywords:** antimicrobial resistance (AMR), cell penetrating peptide (CPP), nucleic acids, antimicrobial peptides, microbial infections

The rise of antimicrobial resistance is a global threat, steadily eroding the effectiveness of existing antibiotics and limiting treatment options. This Research Topic brings together recent advancements in nucleic acid- and peptide-based approaches exploited for the control of infectious pathogens. More specifically, aspects such as mechanisms of action and assessment of effects on bacterial proliferation and virulence for a range of pathogens are described here.

The majority of the original research articles in this Research Topic are related to peptide-based approaches, which is likely a reflection of the larger number of research groups working with these molecules. Giugliano et al. reported the use of pantinins (isolated from the venom of the *Pandinus imperator* scorpion) as a promising class of peptides that exert a membranolytic antimicrobial action against various clinical isolates of *Klebsiella pneumoniae*. This same microorganism was used as a target for 8 different peptides of human, insect, or synthetic origin (Hanstein et al.). In another study, the D-Bac8c^2, 5Leu^ peptide was shown to significantly reduce the viability of monospecies and polymicrobial biofilms of *Staphylococcus aureus* and *Pseudomonas aeruginosa* (Shahrour et al.). Furthermore, D-Bac8c^2, 5Leu^-loaded hydrogel formulations provided sustained drug release and an enhanced antimicrobial activity. Another peptide, bacillomycin D, isolated from *Bacillus amyloliquefaciens*, also showed potent activity against the fungal pathogen *Fusarium graminearum* (Liu et al.). In a related work, another antimicrobial lipopeptide and phytohormone complex powder prepared from *B. amyloliquefaciens* was also assessed for antimicrobial activity, aiming to treat Citrus Huanglongbing (HLB) disease (Ding et al.). Two strategies were assessed against extensively drug-resistant (XDR) Gram-negative pathogens. The first one involved a novel class of anthrocolins, with potent antimicrobial activities reported to interfere with amino acid metabolism in *Pseudomonas aeruginosa* (Yang et al.), and another comprising the synergistic action between peptide-neomycin conjugates and polymyxin B (Story et al.).

In addition, two contributions to this Research Topic describe advances in nucleic acid-based approaches. In one of them, engineered hammerhead ribozymes were directed against the mRNA of an essential gene of *Escherichia coli* (Miszkiewicz-Golec et al.). This promising approach allowed inhibition of bacterial growth by up to 70% over 24 h and enhanced tetracycline efficacy 2- to 4-fold. To assess their action, the ribozymes were incorporated into bacterial plasmids for future applications; however, a direct delivery method for the ribozymes to the bacteria still needs to be devised. In another study, Mendez et al. evaluated whether antisense phosphorodiamidate morpholino oligomers retain activity against biofilms of the *Burkholderia cepacia* complex (Bcc). These same oligomers had been previously conjugated with a peptide for delivery into bacterial cells and were active against planktonic bacteria. Using the same strategy, the authors concluded that the oligomer delivery and antimicrobial activity are still present to some extent within biofilms. In the future, *in vivo* studies should be performed to understand if these oligomers could become viable alternatives for the treatment of conditions such as pulmonary Bcc infections.

Finally, this Research Topic also includes two reviews. One, from Wojciechowska, described the application of endolysins, bacteriophage-encoded peptidoglycan hydrolases, and membrane-active peptides as next-generation antibacterial agents against Gram-negative bacteria; while another by Su et al. provided a general overview of antimicrobial peptides, including their original sources (e.g., animal- or plant-derived), along with their structure, optimization strategies, and main applications.

Collectively, these studies highlight the potential of nucleic acid- and peptide-based strategies to address resistant strains or polymicrobial infections via unique mechanisms, and will certainly assist in tackling the rise of antimicrobial resistance. We would like to thank the authors and co-authors of these articles for their valuable contributions to this field, and hope that the readers will enjoy this Research Topic.

